# Early Comprehensive Physiotherapy in Reconditioning Postoperative Emphysematous Pyelonephritis Patients: A Case Report

**DOI:** 10.7759/cureus.77967

**Published:** 2025-01-25

**Authors:** Mahek R Mohani, Gauri Bhutada

**Affiliations:** 1 Cardiovascular and Respiratory Physiotherapy, Ravi Nair Physiotherapy College, Datta Meghe Institute of Higher Education and Research, Wardha, IND

**Keywords:** emphysematous pyelonephritis, pcnl, physiotherapy, post-operative pulmonary complications, pulmonary rehabilitation, therapeutic interventions

## Abstract

In this case report, a 69-year-old man with a 25-year history of type 2 diabetes mellitus was admitted with severe symptoms like flank pain radiating to the groin, high-grade fever, hematuria, and respiratory symptoms, including shortness of breath and a productive cough. After a complete evaluation, he was diagnosed with emphysematous pyelonephritis (EPN), and to treat this condition, the patient underwent a surgery called percutaneous nephrolithotomy (PCNL), which is used to remove kidney calculi. EPN is a condition characterized by severe infection of the kidney tissue. After the surgery, the patient started experiencing respiratory distress, due to which he was referred to the cardiorespiratory physiotherapy department for further treatment. On the initial assessment, it was found that he had respiratory complications like reduced chest expansion, crackles heard in the lower part of his left lung, and moderate to severe breathlessness (Modified Medical Research Council grade III). A two-week physiotherapy program was planned for the patient, which focused on managing pain, improving chest expansion, clearing the airways, and increasing overall mobility. A few physiotherapy interventions were used, such as transcutaneous electrical nerve stimulation (TENS), deep breathing exercises, the active cycle of breathing technique, and early mobilization. After two weeks of rehabilitation, there was increased chest expansion and reduced shortness of breath and pain. He could also walk during the six-minute walk test (6MWT), which showed improved functional capacity. This case highlights the vital role of early physiotherapy in managing post-surgical complications and promoting early recovery in patients who have undergone PCNL surgery for EPN.

## Introduction

The kidney's basic structural unit, the nephron, is made up of the tubule and glomerulus. Around a million nephrons containing glomerulus and tubule are present in both kidneys. The function of the glomerulus is the filtration of blood, resulting in the formation of ultrafiltrate. The tubule performs the function of concentrating or degrading several substances to create urine. Therefore, these activities help maintain the body's water, electrolytes, and waste products, but the kidneys also play a role in regulating blood pressure, the metabolism of calcium, and the production of red blood cells [1]. Chronic kidney disease has an impact on 10-13% of people and gets worse over time. It often shows no symptoms until it reaches later stages. The doctor aims to slow down kidney problems, handle complications, and get ready for kidney replacement therapy [2].

Emphysematous pyelonephritis (EPN) is a serious, often fatal kidney infection linked to gas production. It is commonly associated with uncontrolled diabetes and urinary tract obstruction. Possible side effects include severe proteinuria, hematuria, and abrupt renal failure [3]. According to the radiological classification, EPN is classified as follows: class 1: the presence of gas in the collecting system; class 2: gas in the renal parenchyma; class 3A: gas extension to the perinephric space; class 3B: gas extension in the pararenal space; and class 4: with bilateral or single kidney involvement [4]. To reduce the mortality risks in EPN patients, conservative management should be the primary strategy [5]. The gold standard for treating kidney stones is percutaneous nephrolithotomy (PCNL) since improvements in optics and stone fragmentation allow for narrower tracts, lower complications, and higher stone clearance rates [6].

Although there is significant treatment morbidity with conventional PCNL, the kidney is successfully healthy and stone-free. To lower the illness of PCNL while achieving an equal stone-free rate, doctors introduced a smaller form of conventional PCNL (mini-PCNL) in 2001 [7]. Over 234 million major surgeries are carried out annually worldwide, with postoperative pulmonary problems being the most frequent serious problem following abdominal surgery [8]. Postoperative lung problems occur at least as often as heart issues during non-heart surgery [9]. These problems increase the risk of dying in the hospital after open-belly surgery [10]. A lung problem that develops after surgery and causes significant illness or dysfunction affecting the course of the initial condition is called a postoperative pulmonary complication (PPC) [11].

Research shows that changing positions from lying flat to sitting up increases the amount of air breathed per minute [12]. Currently recommended by the American College of Chest Physicians and the European Society of Thoracic Surgeons, physiotherapy is an essential component of postoperative care after thoracic surgery. Accelerating recovery and decreasing hospital stays is crucial for enhanced recovery protocols (ERPs) [13].

## Case presentation

Patient information

The chief complaints of a 69-year-old man who contacted the medicine department were severe discomfort radiating to his groin area, a high-grade fever with chills, episodes of vomiting, dyspnea, nausea, and frequent urination along with blood for a month. The patient was a known case of type 2 diabetes mellitus for 25 years. Following examinations and investigations, the patient was given a diagnosis of emphysematous pyelonephritis and advised to have a PCNL procedure. The patient was referred to the cardiorespiratory physiotherapy department for additional care after the recommended procedure. Table 1 includes a description of the postoperative symptoms the patient was experiencing.

**Table 1:** Description of postoperative symptoms. ADLs: activities of daily living; MMRC: Modified Medical Research Council.

Symptoms	Onset	Duration	Type	Aggravating factors	Relieving factors
Flank pain	Gradual	1 month	Severe, radiating	Sitting, walking, ADLs	Medications
Fever	Sudden	2 weeks	High fever with chills	ADLs	Rest, medications
Cough	Gradual	1 month	Productive cough with whitish sputum	Exertion, physical activity	Rest, medications
Dyspnea	Gradual	1 month	Progressive (MMRC grade 3)	ADLs, exertion, fever	Rest, medications

Clinical findings

Informed consent was taken from the patient before examination. Throughout the general examination, the patient remained cooperative, aware, and hemodynamically stable. With a body mass index (BMI) of 24.61 kg/m2, the patient was mesomorphic. Upon examination of the abdomen, a bandage covering the left kidney's posterolateral lumbar area was observed, accompanied by a grade III tenderness surrounding the suture site.

During the respiratory examination, the chest was found to be barrel-shaped, with supraclavicular hollowness and retraction. At the nipple and xiphisternum levels, there was less chest enlargement. Reduced left-sided chest symmetry was noted. Dullness was felt in the supra and inframammary areas, more of the right lung upon percussion. Crackles were audible in lower lung zones, more on the right side during auscultation.

**Figure 1:**
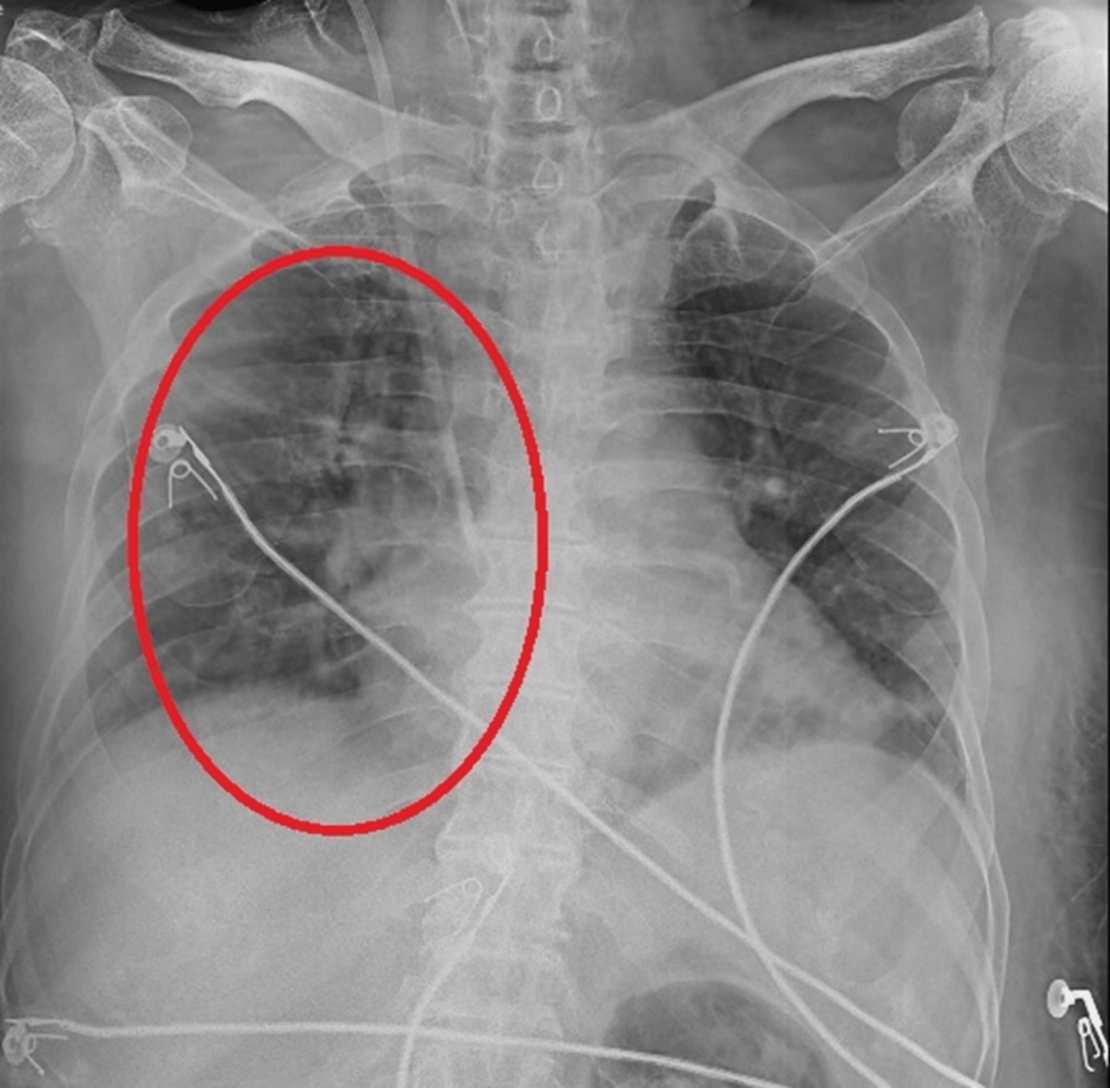
Pre-physiotherapy rehabilitation X-ray.

Figure 1 depicts the X-ray before physiotherapy treatment, which basically shows increased opacity in the lower lung fields, particularly more on the right side, and the absence of clear lung markings in some areas.

**Figure 2:**
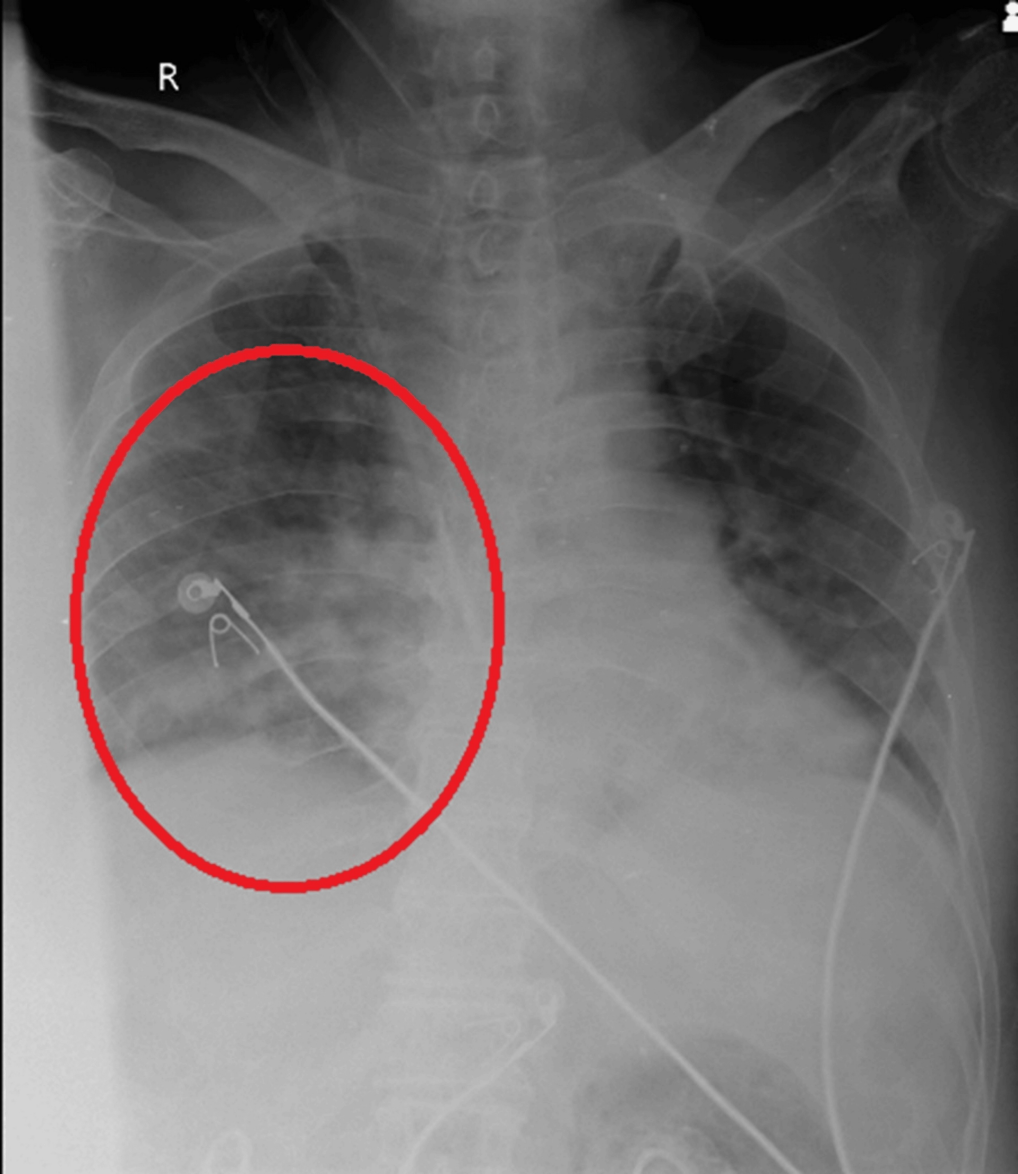
Post-physiotherapy rehabilitation X-ray.

Figure 2 depicts the X-ray after physiotherapy rehabilitation, which depicts decreased opacity and clear bronchovascular markings present.

Physiotherapy intervention

The physiotherapy intervention begins by educating the patient and the family members about the condition, its complications, and the role of physiotherapy in improving or maintaining the condition. The two-week rehabilitation protocol designed for the patient is mentioned in Table 2.

**Table 2:** Problem list, goals, and intervention. TENS: transcutaneous electrical nerve stimulation; ACBT: active cycle of breathing technique; ADLs: activities of daily living; DVT: deep vein thrombosis.

Problem list	Goals	Intervention
Lack of education	To educate patient and family	Educating family and patient about the condition and role of physiotherapy protocol
Pain	To reduce pain at suture site	TENS, relaxation techniques, positioning, splinted coughing/huffing
Reduced chest expansion	To increase chest expansion	Deep breathing exercises, thoracic expansion exercises
Cough with sputum	To remove secretions	Postural drainage, ACBT, percussion, and vibration
Dyspnea	To reduce dyspnea	Dyspnea relieving positions
Reduced mobility	To improve mobility	Bed mobility exercises, assisted transfers, walking aids
Reduced core, lower back, and abdominal muscle strength	To enhance core, lower back, and abdominal muscle strength	Strengthening exercises: isometric exercises, progression to resisted exercises
Forward neck posture	To correct the postural abnormality	Postural correction exercises: chin tucks, scapular elevation, depression
Reduced efficacy of walking	To improve walking efficiency	Ambulation
Reduced functional independence	To improve functional independence	ADLs training
To prevent secondary complications	DVT and bed sore prevention	Ankle pumps, compression stockings, limb elevation, positioning

**Figure 3:**
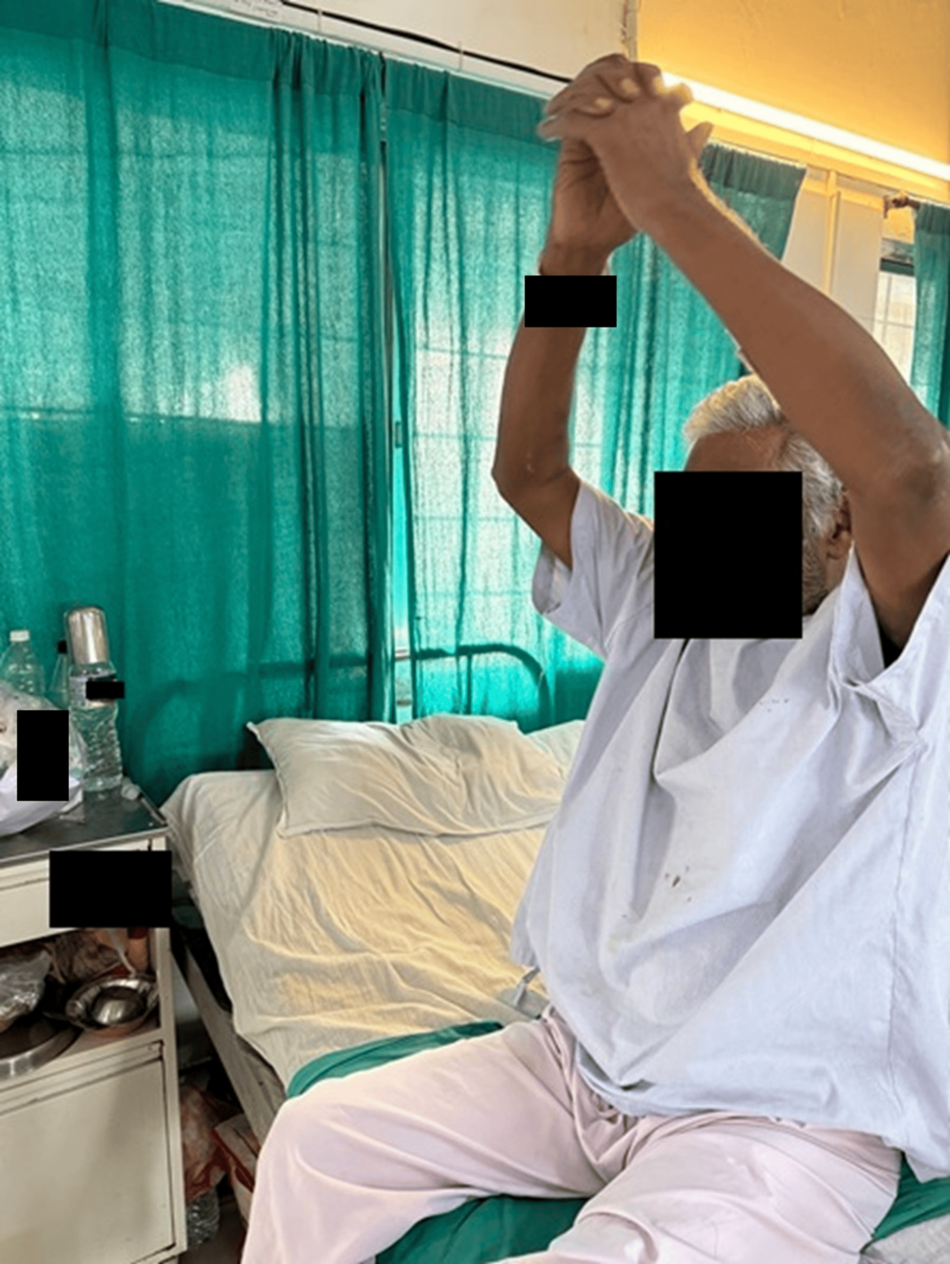
The patient performing thoracic expansion exercises for enhancing chest expansion.

The patient performing thoracic expansion exercise is depicted in Figure 3.

Outcome measures

The outcome measures were recorded on the day he was referred and after two weeks of rehabilitation. Various outcome measures were used, and the pre and post-rehabilitation scores are mentioned in Table 3. The post-rehabilitation values of pain significantly reduced, along with improved chest expansion, Short Form 36 (SF-36) scores, and reduced dyspnea.

**Table 3:** Outcome measures. NPRS: numerical pain rating scale; BBS: Berg Balance Scale; MMRC: Modified Medical Research Council; 6MWT: six-minute walk test; KFT: kidney function test; SF-36: Short Form 36.

Outcome measures	Pre-rehabilitation (day of referral)	Post-rehabilitation (after 2 weeks)
NPRS		
On rest	4/10	2/10
On activity	9/10	5/10
Chest expansion	Upper lobe - 102-100 = 2 cm; middle lobe - 99-97 = 2cm; lower lobe - 99-96 = 3 cm	Upper lobe - 106-104 = 2 cm; middle lobe - 105-102 = 3 cm; lower lobe - 103-99 = 5 cm
Dyspnea (MMRC)	Grade III	Grade I
6MWT	220 m	380 m
BBS	40	48
KFT	Urea = 159 mg/dL; creatinine = 6.5 mg/dL; sodium = 127 mEq/L; potassium = 5.2 mEq/L	Urea = 60-90 mg/dL; creatinine = 2.0-3.5 mg/dL; sodium = 133-137 mEq/L; potassium = 4.0-4.5 mEq/L
SF-36		
Restrictions in physical activities due to health complications	35	60
Limitations in social activities because of physical or emotional problems	40	55
Limits in usual role activities due to physical health problems	30	55
Bodily pain	25	50
Overall mental health	40	65
Limitations in typical role activities because of emotional problems	35	60
Energy and fatigue	30	55
Perceptions toward health	35	60

## Discussion

This case report presents a case of a 69-year-old man diagnosed with emphysematous pyelonephritis (EPN), who came with respiratory symptoms to the cardiorespiratory department for which a two-week protocol was specially designed based on his complaints and examination results. The outcome measures like the numerical pain rating scale, Modified Medical Research Council grading, Berg Balance Scale (BBS), and chest expansion all showed improvements in the post-rehabilitation scores, which eventually led to reduced pain, increased chest expansion, improved mobility, enhanced functional capacity, and improved physical and mental function of the patient.

One of the primary goals of physiotherapy post surgery is to prevent postoperative pulmonary complications (PPCs) like atelectasis and pneumonia. Diminished lung volumes, disrupted lung muscle function, lowered mucociliary clearance, and pain-related inhibition of respiratory muscles are the causes of pulmonary problems, such as pneumonia and severe atelectasis [14]. Breathing exercises can stop mild atelectasis during the first 24 hours following surgery [15]. According to reports, preoperative education and breathing exercise training alone can reduce PPC risk by 75% relative to 20% absolute [16]. Silva et al. state that getting patients up and moving after surgery, along with physical therapy, helps prevent postoperative lung problems [17]. Following thoracic surgery, chest physical therapy is advised for airway clearing to increase mobility, aid with secretion clearance, and improve reduced lung volume, all of which lower the incidence of PPC. To help patients remove sputum from their airways, chest physical therapy uses manual chest percussion, active cycle of breathing technique (ACBT), and deep breathing exercises [18]. This study supports the findings, with the patient showing substantial improvements in chest expansion and reduced dyspnea after just two weeks of rehabilitation.

Usually after thoracic surgeries, there is a lot of pain that has been referred to as being among the most painful types of surgical incisions [19]. According to Freynet et al., being in pain makes it difficult to cough and take deep breaths, resulting in reduced lung capacity and a higher possibility of re-intubation. Postoperative discomfort after thoracic surgery can be safely and effectively relieved through transcutaneous electrical nerve stimulation (TENS), which accelerates the healing process and enhances outcomes [20].

In addition to addressing pain, it is also advised that early ambulation could be employed as a procedure to avoid PPC [21]. Schaller et al., through their study, indicated that patients who engage in early postoperative mobilization exhibit faster recovery times, reduced hospital stays, and fewer complications [22]. This was evident in the current case, as reflected in the six-minute walk test (6MWT), which increased from 220 m to 380 m. Overall, the interventions used in this case study were aligned with the findings of studies that support the role of physiotherapy in enhancing postoperative recovery.

## Conclusions

This case report underscores the importance of early physiotherapy intervention in the postoperative management of EPN following PCNL. The tailored rehabilitation protocol significantly improved the patient's respiratory function, pain management, mobility, and overall recovery. These findings highlight the efficacy of integrating physiotherapy in enhancing patient outcomes and reducing complications in complex cases involving severe renal infections and comorbidities like diabetes mellitus.
